# The effectiveness of a stage-based lifestyle modification intervention for obese children

**DOI:** 10.1186/s12889-018-5206-2

**Published:** 2018-03-01

**Authors:** Nor Baizura Md. Yusop, Zalilah Mohd Shariff, Ting Tzer Hwu, Ruzita Abd. Talib, Nicola Spurrier

**Affiliations:** 1 Department of Nutrition and Dietetics, Faculty of Medicine and Health Sciences, Universiti Putra Malaysia, 43400 Serdang, Selangor Malaysia; 2 Department of Pediatrics, Faculty of Medicine and Health Sciences, Universiti Putra Malaysia, 43400 Serdang, Selangor Malaysia; 3 Department of Nutrition & Dietetics, Faculty of Allied Health Sciences, Universiti Kebangsaan Malaysia, Jalan Raja Muda Abdul Aziz, 50300 Kuala Lumpur, Malaysia; 4 Department of Paediatrics and Child Health, Flinders University and Flinders Medical Centre, Bedford Park, South Australia 5042 Australia

**Keywords:** Obese children, Lifestyle intervention, Dietary intake, Body composition, Physical activity

## Abstract

**Background:**

Interventions that encompass behavioural modifications of dietary intake and physical activity are essential for the management of obesity in children. This study assessed the effectiveness of a stage-based lifestyle modification intervention for obese children.

**Methods:**

A total of 50 obese children (7–11 years old) were randomized to the intervention group (IG, *n* = 25) or the control group (CG, *n* = 25). Data were collected at baseline, at follow-up (every month) and at six months after the end of the intervention. IG received stage-based lifestyle modification intervention based on the Nutrition Practice Guideline for the Management of Childhood Obesity, while CG received standard treatment. Changes in body composition, physical activity and dietary intake were examined in both the intervention and control groups.

**Results:**

Both groups had significant increases in weight (IG: 1.5 ± 0.5 kg; CG: 3.9 ± 0.6 kg) (*p* < 0.01) and waist circumference (IG: 0.1 ± 0.5 cm; CG: 2.2 ± 0.7 cm) (*p* < 0.05), but the increases were significantly higher in CG than IG. Body Mass Index (BMI)-for-age z scores decreased significantly in IG (− 0.2 ± 0.0, p < 0.01) but not in CG. The physical activity of the IG significantly increased (0.44 ± 0.13) compared with that of CG (− 0.28 ± 0.18), and the difference in mean change between groups was statistically significant (*p* < 0.05). Dietary intake was not significantly different between the two groups. However, calorie and carbohydrate intake decreased significantly in both groups.

**Conclusions:**

A stage-based intervention that modified dietary and physical activity behaviour may be effective in weight management for obese children.

**Trial registration:**

NCT03429699 retrospectively registered 9 February 2018.

## Background

The number of infants and young children (< 5 years) worldwide who are overweight or obese has steadily increased from 32 million in 1990 to 41 million in 2016. Furthermore, if current trends continue, the count will increase to 70 million by 2025 [1]. The rise in Body Mass Index (BMI) among children aged 5 to 19 years has accelerated for both sexes in East and South Asia and for boys in Southeast Asia. The global age-standardized prevalence of obesity increased from 0·7% (0·4–1·2) in 1975 to 5·6% (4·8–6·5) in 2016 in girls and from 0·9% (0·5–1·3) in 1975 to 7·8% (6·7–9·1) in 2016 in boys [2]. In Malaysia, the Fourth National Health and Morbidity Survey (NHMS IV)(2015) reported that the prevalence of obesity among children aged 0–18 years was 11.9% [3]. Recent data from the Nutrition Survey of Malaysian Children (SEANUTS Malaysia) showed that the prevalence rates of overweight and obesity for children 6 months to 12 years old were 9.8% and 1.8%, respectively [4]. Although childhood obesity is not defined as a disease, obese children are at risk of metabolic syndrome [5], severe obesity in adulthood and health problems later in life [6].

The increasing prevalence of overweight and obesity in children necessitates effective treatment strategies to prevent the development of chronic diseases in the future. An effective treatment comprising behavioural modification to manage dietary intake and physical activity produced a clinically significant weight reduction in obese children [7], defined as weight loss of at least 0.5 BMI z-score units [8]. The transtheoretical model (TTM), by Prochaska & DiClemente [9], has been frequently used in behavioural change interventions related to smoking, emotional distress, alcohol abuse, weight loss and mammography screening [10–12]. The central organizing construct of the model consists of the stages of change (SOC), which represent the process, from precontemplation to maintenance, that individuals undergo to change their behaviour for health improvement [9]. The SOC process has been used in several childhood obesity interventions to reduce intake of fat, increase intake of fruits and vegetables and increase duration of physical activity to promote weight loss [13–15].

With the increasing prevalence of childhood obesity in Malaysia, an effective treatment strategy is required to prevent adverse health outcomes. To date, only a few studies have been conducted on childhood obesity intervention in Malaysia [16, 17]. The present study used a stage-based approach to modify diet and physical activity behaviours of obese children for weight management. This study assessed the effectiveness of a stage-based lifestyle modification intervention on body composition, physical activity and dietary intake among obese children.

## Methods

### Study design and participants

Out of 284 obese children aged 7–11 years old from five primary schools in three districts of Selangor, 50 children were recruited into the study. Obesity was defined as a BMI-for-age z score greater than + 2 SD [18]. Obese children diagnosed with chronic asthma, diabetes mellitus, psychiatric disorders (e.g., schizophrenia, severe autism or mental retardation), or other serious medical conditions; those who were taking medications that might promote weight gain or weight loss; and those who were already participating in any weight management programme were excluded.

After the screening process at schools, an invitation letter, the child’s BMI-for-age growth chart and a study information sheet were given to the parents/caregivers of obese children to encourage participation. A brief explanation of the study was also given to the parents through a phone call. The parents/caregivers who expressed an interest in participating in the study were scheduled for a recruitment appointment. After the parents/caregivers completed the consent form, the participants were age matched and randomized using a permuted block method, with a block sizes of four, into either IG or CG [19]. To guarantee *allocation* concealment, we had the *randomization* carried out *by an independent third party*.

A significant mean difference of − 0.25 and a standard deviation of 0.21 for BMI z-scores over six months were used in the effect size formula, giving a large value of 1.19 [20]. Therefore, t*he sample size required to ensure a minimum predictive power of 80% with a 0.05 probability of type I error, assuming a 30% drop-out rate, was determined to be 15 obese children per group. Calculation was performed using the* mean difference between the two groups [21]*.* The intervention study was conducted at the Dietetic Clinic, Faculty of Medicine and Health Sciences, Universiti Putra Malaysia (UPM). Ethical approval and permission to conduct the intervention were obtained from the Medical Research Ethics Committee of UPM and the Ministry of Education Malaysia, respectively.

### Treatments

The treatments for the two groups are summarized in Table 1. Participants in IG received a stage-based lifestyle modification that comprised several activities, including nutrition counselling, aerobic sessions, a hands-on activity (healthy food preparation) and ‘Sharing is Caring’. The Nutrition Practice Guideline (NPG) for the Management of Childhood Obesity, which comprises recommendations for assessment of nutritional status, determination of energy requirements, dietary prescriptions and physical activity modifications, was used [22]. Participants’ SOC for dietary and physical activity behaviours were determined before any information was given to ensure that the selected educational topic was tailored to the participant’s current stage. Several educational tools matching each participant’s current SOC were used. Nutritional counselling lasted for one hour, and at least two goals for diet and physical activity were determined at the end of the session. The researcher (Nor Baizura, M.Y.), a trained dietitian, provided nutrition counselling to parents/caregivers and children. An aerobic session was conducted to increase participants’ motivation levels and encourage them to be more active, and a hands-on activity was conducted with the aim of increasing the participants’ knowledge about healthy food preparation.

**Table 1:** Summary of treatments received by the two groups

Week	Intervention group						Control group
	Nutritional counselling NPG for the Management of Childhood Obesity			Additional activities			Nutritional counselling Standard treatment for the management of childhood obesity
	Topic	Contents/activities	Behavioural change technique(s)	Aerobic session (parents & children)	Hands-on activity (children)	Sharing is Caring (parents)	
0	Increase awareness	• Increase awareness of childhood obesity • Discuss the risks and complications of obesity • Discuss the pros and cons of weight reduction • List and discuss barriers to modification of fat/fruit/vegetable intake and PA • Establish readiness to change	Consciousness raising Decisional balance				1. Decrease daily caloric intake by 500–1000 kcal from current level 2. Macronutrient distribution (carbohydrates: 50–60%; protein: 15–20%; fat: 25–30%) 3. Eat more fruits and vegetables
1–4	Set goals	• Discuss energy balance • Provide personally tailored information about recommended fat/fruit/vegetable intake and PA • Set specific goals for changes in fat/fruit/vegetable intake and PA (e.g., consume less fried food) • Create a plan for fat/fruit/vegetable intake and PA change	Self-re-evaluation Environmental re-evaluation Goal setting	Low-intensity aerobic exercise			
5–8	Be positive	• Encourage the family/social group to support the child • Focus on self-management skills • Provide personalized information using self-help materials • Provide guidance on the duration, intensity, and frequency of activities (only PA)	Self-liberation Stimulus control				
9–12	Identify challenges	• Review behaviours that have helped the child adhere to fat/fruit/vegetable intake modification • Praise the child or adolescent for being physically active		Medium-intensity aerobic exercise			
13–16	Be consistent	• Focus on problem solving • Teach the child how to identify and address relapse • Maintain diet and PA changes • Recommend more challenging dietary changes if the patient and family are motivated	Problem solving Reinforcement Management				
17–20	Situation management	• Encourage the parents and child to anticipate and plan for potential difficulties/barriers (e.g., maintaining dietary changes on vacation, at school, and at restaurants)	Problem solving Counterconditioning	High-intensity aerobic exercise	Healthy food preparation.	Sharing experiences	
21–24	Maintaining changes	• Encourage the patient to ‘recycle’ if he/she has a lapse or relapse • Identify strategies to help the patient remain physically active • Increase his/her confidence in his/her ability to remain physically active • Encourage family/social group to support the child	Helping relationship Dramatic relief Environmental evaluation				

Each participant was required to participate in three sessions of aerobic exercise on weekends (once every two months). The aerobic exercise sessions were conducted by a professional instructor, and each session lasted for two hours. The first hour focused on aerobic exercise. The movements used during the aerobic exercises were easy and suitable for participants to follow. In addition, participants were allowed to rest for 5 min after each 15 to 20 min of aerobic exercise. In the second hour of the session, the instructor demonstrated activities or movements that would be feasible and enjoyable to perform at home as indoor exercise. The activities involved several movements that do not require special tools, only balloons and mineral water bottles. During the demonstration, the instructor also explained the benefits of movements, such as increased muscle strength and energy usage, to motivate participants to perform physical activities.

A ‘Sharing is Caring’ session was to encourage parents/caregivers to share their experiences during the intervention period. The session lasted for one and a half hours. Before each session, the researcher explained the purpose of the session to the parents/caregivers and reminded them that there are no incorrect responses and that the session would be audio recorded. The topics discussed were (1) parents’ experiences with the dietitian, (2) parents’ experiences in managing their child’s lifestyle modifications, (3) strengths and weaknesses of the intervention and (4) their plan to maintain the lifestyle changes.

The CG received standard treatment based on current practices by most dietitians in Malaysia for weight management of obese children [22]. Nutrition counselling was provided by dietitians but was not delivered on the basis of SOC as it was in the IG. During nutrition counselling, participants were assessed and informed about their nutritional status, after which they were given advice on dietary intake and physical activity. Participants were advised to reduce their intake of fats and sweetened drinks, increase their intake of fruits and vegetables, increase their physical activity and reduce their sedentary activity, but they were not instructed specifically about the quantities of food to consume. The nutritional counselling was conducted for half an hour, and only a related brochure on childhood obesity was disseminated.

### Outcome measures

Measurements of body composition, physical activity and dietary intake were conducted at baseline, at follow-up visits (every four weeks) and at 24 weeks after the end of the intervention. Body weight and height were measured using a SECA 701 digital column weighing scale (SECA Vogel and Halke Gmbh & Co., Germany). BMI-for-age z-score was calculated using the WHO AnthroPlus software (http://www.who.int/growthref/tools/en/), and the WHO 2007 growth reference was used to determine the BMI-for-age z-scores of participants [18]. Waist circumference (WC) was measured using a fibreglass tape (SECA 201 measuring tape, SECA Vogel and Halke Gmbh & Co., Germany) at the highest point of the iliac crest. Body fat percentage (to the nearest 0.5%) was calculated using the equation of Slaughter et al. [23], which required measurement of skinfold thickness at two sites (triceps and subscapular). A Lange skinfold calliper (Cambridge Instrument, Cambridge, MA, USA) was used to measure the skinfolds.

The Physical Activity Questionnaire for Older Children (PAQ-C) was used to assess physical activity. The questionnaire is a self-administered instrument based on seven-days’ recall and comprises 10 items. The answer for each item ranges from the lowest-activity response and progresses to the highest-activity response. The lowest-activity response was scored as one, and the highest-activity response was scored as five. Participants were classified into the categories of low, moderate and high physical activity, defined by mean total scores ranging from 1.00 to 2.33, 2.34 to 3.66 and 3.67 to 5.00, respectively [24].

Dietary intake was ascertained using a three-day food record at every visit. Participants and parents were taught about portion size estimation and household measurement to fill in the three-day food record. Pictures from the Atlas of Food Exchanges and Portion Sizes (food album) [25], a set of household measurement tools (glass, soup bowl, plate, cup, teaspoon and tablespoon) and food models were used to guide parents in estimating portion sizes. At every visit, participants submitted their written food record to the dietitian and were interviewed to assure the completeness of the records. Dietary data were analysed for energy and nutrients (carbohydrate, protein, fat, saturated fatty acids (SFA), dietary fibre and sugar) using the Nutritionist Pro™ software version 2.5 (First Data Bank, USA, 2005).

### Confounders

Demographic and socioeconomic variables, including age, gender, ethnicity, parents’/caregivers’ education level and current estimated household income were obtained through a parent-administered questionnaire.

### Statistical analysis

Data were analysed using SPSS Statistics v21.0 (SPSS Inc., 2010, Chicago, Illinois). The statistical analysis was performed on a per-protocol basis: all participants who fulfilled the protocol in terms of the eligibility and intervention were included in the analysis to evaluate group differences in outcome measures at baseline and at 24 weeks. Between-group differences six months after the intervention were measured with an independent-sample t-test for continuous data and Pearson’s chi-squared test for categorical data. A paired t-test was conducted to assess changes in outcome variables within each group (IG and CG) and between the baseline and six-month post-intervention measurements.

## Results

**Fig. 1:**
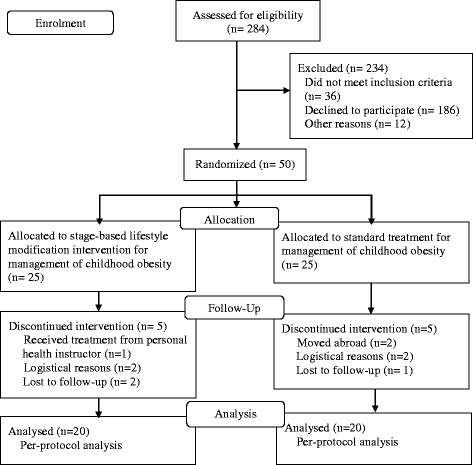
Flow diagram of stage-based lifestyle modification intervention compared with standard management for childhood obesity

Figure 1 shows the flow diagram of the stage-based lifestyle modification intervention compared with standard management for childhood obesity. At the end of 24 weeks, 40 participants completed the study, yielding a total dropout rate of 20%. Five participants in IG withdrew because they were receiving treatment from a personal health instructor (1), for logistical reasons (2) or by being lost to follow-up (2), while five participants in CG were excluded because of moving abroad (2), logistical reasons (2) or loss to follow-up (1).

The participants comprised 52.5% (21) boys and 47.5% (19) girls (Table 2). The mean age of the participants was 9.8 ± 1.2 years. At baseline, there was no significant difference between the groups in any of the demographic and socioeconomic characteristics. Table 3 shows body composition, PAQ-C score and dietary intake in each group at baseline and after 24 weeks of intervention. None of the measurements was significantly different between the two groups at baseline. After the intervention, the mean changes in weight (IG: 1.5 ± 0.5 kg, CG: 3.9 ± 0.6 kg) and WC (IG: 0.1 ± 0.5 cm, CG: 2.2 ± 0.7 cm) between IG and CG were statistically significant (*p* < 0.05). Although weight increased significantly in the IG (1.5 ± 0.5 kg, *p* < 0.01), BMI-for-age z-score significantly decreased after the intervention (− 0.2 ± 0.0, *p* < 0.01) at 24 weeks.

**Table 2:** Demographic and socioeconomic characteristics of the participants

Variable	All	Intervention	Control	χ^2^ value	*P* value
	(*n* = 40)	(*n* = 20)	(*n* = 20)		
	n (%)	n (%)	n (%)		
Age (years)					
7–9	17 (42.5)	8 (20.0)	9 (22.5)	0.102	0.749
10–11	23 (57.5)	12 (30.0)	11 (27.5)		
Gender					
Boys	21 (52.5)	11 (27.5)	10 (25.0)	0.100	0.752
Girls	19 (47.5)	9 (22.5)	10 (25.0)		
Ethnicity					
Malay	38 (95.0)	19 (47.5)	19 (47.5)	0.000	1.000
Non-Malay	2 (5.0)	1 (2.5)	1 (2.5)		
Father’s educational level					
Primary or secondary education	17 (42.5)	9 (22.5)	8 (20.0)	0.102	0.749
Tertiary education	23 (57.5)	11 (27.5)	12 (30)		
Mother’s educational level					
Primary or secondary education	17 (42.5)	8 (20.0)	9 (22.5)	0.102	0.749
Tertiary education	23 (57.5)	12 (30)	11 (27.5)		
Current estimated household income					
RM 2001 to RM 5000	9 (22.5)	4 (10.0)	5 (12.5)	0.143	0.705
> RM 5000	31 (77.5)	16 (40.0)	15 (37.5)

**Table 3:** Body composition, physical activity and dietary intake in each group at baseline and after 24 weeks of intervention

Characteristics	IG		p^a^	CG		p^a^	Changes		p^b^
	Baseline	24 weeks		Baseline	24 weeks		Intervention	Control	
	(*n* = 20)	(*n* = 20)		(*n* = 20)	(*n* = 20)		(*n* = 20)	(*n* = 20)	
							(mean ± S.E.)	(mean ± S.E.)	
				(mean ± S.D.)	(mean ± S.D.)				
	(mean ± S.D.)	(mean ± S.D.)							
Body composition									
Height (cm)	139.6 ± 7.7	142.5 ± 8.0	0.000**	139.4 ± 6.8	142.6 ± 7.5	0.000**	2.9 ± 0.3	3.2 ± 0.4	0.504
Weight (kg)	55.8 ± 12.2	57.3 ± 11.4	0.004*	55.6 ± 9.6	59.5 ± 11.2	0.000**	1.5 ± 0.5	3.9 ± 0.6	0.004*
BMI-for-age	3.2 ± 0.6	3.0 ± 0.5	0.000**	3.4 ± 0.8	3.3 ± 0.8	0.075	−0.2 ± 0.0	−0.1 ± 0.1	0.060
WC (cm)	87.5 ± 8.2	87.6 ± 7.5	0.891	86.7 ± 6.7	88.9 ± 7.2	0.004*	0.1 ± 0.5	2.2 ± 0.7	0.018*
Fat percentage (%)	42.9 ± 8.4	43.4 ± 8.3	0.262	42.9 ± 6.6	44.9 ± 6.5	0.007**	0.6 ± 0.5	2.0 ± 0.7	0.098
Physical activity									
PAQ-C score	1.93 ± 0.54	2.38 ± 0.12	0.003**	2.04 ± 0.80	2.01 ± 0.90	0.89	0.44 ± 0.13	−0.28 ± 0.18	0.045*
Dietary intake									
Calorie (kcal)	1955 ± 692	1370 ± 337	0.001**	1855 ± 670	1392 ± 268	0.013*	− 584.8 ± 149.7	− 463 ± 168	0.590
Carbohydrate (g)	258.4 ± 85.6	172.5 ± 58.2	0.000**	242.1 ± 92.9	168.8 ± 35.3	0.009**	−85.9 ± 19.4	−73.3 ± 25.2	0.973
% kcal	54.2 ± 8.0	49.8 ± 7.7	0.107	52.9 ± 10.3	48.9 ± 7.4	0.218	−4.4 ± 2.6	−4.0 ± 3.1	0.926
Protein (g)	82.3 ± 54.1	54.7 ± 12.7	0.024*	87.4 ± 48.4	60.4 ± 30.7	0.062	−27.6 ± 11.3	−27.0 ± 13.6	0.694
% kcal	15.9 ± 5.4	16.2 ± 2.4	0.846	19.3 ± 9.8	16.9 ± 5.7	0.384	0.3 ± 1.4	−2.4 ± 2.7	0.382
Fat (g)	72.3 ± 34.4	44.6 ± 13.1	0.004**	66.3 ± 33.8	52.1 ± 17.9	0.151	−27.7 ± 8.3	−14.2 ± 9.5	0.291
% of kcal	32.0 ± 8.2	29.1 ± 4.7	0.188	31.4 ± 9.0	33.3 ± 7.4	0.559	−3.0 ± 2.2	1.9 ± 3.1	0.213
SFA (g)	19.1 ± 11.6	11.8 ± 7.5	0.027*	15.7 ± 10.3	10.4 ± 5.6	0.084	−7.3 ± 3.0	−5.3 ± 2.9	0.063
% kcal	8.2 ± 0.9	6.2 ± 0.9	0.178	7.2 ± 2.8	6.4 ± 6.0	0.655	−2.1 ± 1.5	−0.77 ± 1.6	0.565
Sugar (g)	40.4 ± 24.4	24.7 ± 6.1	0.011*	41.0 ± 30.8	24.2 ± 11.7	0.032*	15.7 ± 5.5	16.9 ± 7.3	0.900
% kcal	8.6 ± 4.4	7.4 ± 2.0	0.280	9.4 ± 7.0	6.9 ± 2.8	0.151	−1.2 ± 1.1	−2.5 ± 1.7	0.503
Dietary fibre (g)	8.3 ± 4.1	9.1 ± 1.9	0.373	7.8 ± 5.0	10.6 ± 4.4	0.022*	0.8 ± 0.8	2.9 ± 1.0	0.149

*IG* intervention group, *CG* control group, *BMI* body mass index, *WC* waist circumference, *PAQ-C* Physical Activity Questionnaire for Older Children, *SFA* saturated fatty acids

PAQ-C scores ranged from one to five (low: 1.00–2.33; moderate: 2.34–3.66; high: 3.67–5.00)

^a^Paired t-test

^b^Independent t-test

*p < 0.05, **p < 0.01

Most participants in IG significantly increased their physical activity level from low (1.93 ± 0.54) to moderate (2.38 ± 0.12), with a mean change of 0.44 ± 0.13 (*p* < 0.01); in CG, however, physical activity score remained low at the end of the intervention, changing only from 2.04 ± 0.80 to 2.01 ± 0.90, with a mean change of 0.28 ± 0.18 (*p* < 0.05). The mean change difference in physical activity score between groups was statistically significant (*p* < 0.05). Energy, carbohydrate (g) and sugar (g) intake decreased significantly in both groups. Protein (g), fat (g) and SFA (g) intake decreased significantly within IG, but dietary fibre intake increased significantly within CG. None of the mean differences in energy or nutrient intake was significantly different between the groups.

## Discussion

The study showed that the stage-based lifestyle modification improved the body composition and physical activity of obese children. The finding that weight gain was significantly lower in IG than in CG was consistent with the findings of trials involving younger children, which generally aimed to reduce the level of overweight by limiting weight gain. Similar to previous studies, the current study also showed a relative reduction in weight gain with continuing growth in treated participants compared with controls, rather than weight loss per se [7, 26]. In addition, a duration of six months for an intervention is ideal for weight gain prevention instead of weight reduction [26].

The WHO growth chart (2007) was used to compare the BMI-for-age z-scores of children of the same age and gender. In monitoring the growth and development of children, we used age- and gender-specific z-scores. The present study found that BMI-for-age z-score was reduced significantly in IG (− 0.2 ± 0.2), and the change was larger than that described in the Cochrane Review, which is − 0.15 kg m^− 2^ for ages 6 to 12 years. The participants in IG maintained their weight while continuing to grow in height [26]. However, there was no significant difference between groups.

We also found that the mean change in WC between IG and CG was statistically significant. CG had a high WC increment, while IG maintained a steady WC through the end of intervention. Sun et al. [27] reported reductions in body fat, truncal fat and WC after ten weeks of one-hour school-based physical activity intervention. They found that the changes in WC did not differ between the diet-restriction and non-diet-restriction groups. Similarly, in the present study, physical activity was significantly different between IG and CG, while none of the mean changes in energy or nutrient intakes was significantly different between the groups. A cross-sectional study by Lee [28] reported that solitary screen time, such as time spent watching television, playing video games, and using the computer, was significantly associated with BMI-for-age z-scores and waist circumference. It seems that waist circumference decreased in response to exercise but not diet or the interaction between the treatments. WC is one of the measures used to estimate visceral adipose tissue, and it has been linked to metabolic disorders in children and adolescents [29]. In addition, WC is more effective than BMI at predicting adiposity and insulin resistance [30]. Preventing increases in WC is important, as the WC of obese children is associated with negative health outcomes later in life [31].

Physical activity is an essential component of any weight management programme to achieve energy balance among obese children [32]. Previous studies reported that structured physical activity programmes were effective in increasing activity-related energy expenditure, which, over a longer period, improves the body composition of obese children [32–34]. At the end of the intervention, most participants in IG (65%) had increased their physical activity level from low to moderate, whereas the physical activity level of the CG group (50%) remained low at the end of the intervention. As part of the treatment, IG received SOC-appropriate advice on physical activity, which educated the IG children and encouraged them to increase their physical activity gradually. Sealy & Farmer [35] found that lifestyle modification according to the SOC of parents improved the dietary intake and physical activity of obese children. Another study by Woods [36] showed that SOC were significantly associated with the participation of young adults in physical activity. Across all the SOC, self-evaluation, self-liberation, counterconditioning and reward processes were the processes of change that were frequently used to alter the children’s behaviour towards greater physical activity.

Instead of routine individual counselling, IG participants were required to attend three aerobic sessions conducted by a professional instructor to increase their motivation levels and encourage them to be more active. Alberga [37] showed that an increase in aerobic exercise was associated with improvements in body image and social competence in obese adolescents, and these psychological benefits were related to improved aerobic fitness. Moreover, IG learned indoor and outdoor activities (i.e., skipping, dancing, walking and cycling). They were encouraged to perform suggested activities as short-duration bouts to increase activity-related energy expenditure. McManus [38] found that an increase in the number of short-duration bouts of movement per day combined with a reduction in periods of rest between bouts of movement is an effective physical activity intervention. A combination of activities such as exercises to promote coordination, exercises devoted to posture and balance, relaxation techniques, rhythm and music, exercises devoted to creative movement, games involving group participation, and practice for back training was effective in increasing the physical activity of obese children if conducted for at least five minutes at a time [32]. An intervention study by Ham [39] implemented a skipping-rope exercise to music to increase physical activity among obese children.

After intervention, none of the mean differences in energy and nutrients was significantly different between the groups. Energy, carbohydrate (g) and sugar (g) intakes\ decreased significantly in IG and CG. The mean difference in carbohydrate intake was positively correlated with the mean difference in energy intake, which indicates that as the intake of energy decreased, the intake of carbohydrates also decreased because calorically dense foods are typically high in carbohydrates [40]. A reduction in total sugar intake contributed to a reduction in carbohydrate intake as well [21]. The results were in line with the findings of Burrows [41], who showed that decreased consumption of energy-dense drinks, particularly sweetened ones, and increased consumption of dietary fibre resulted in the reduction of total energy intake. Similarly, after two years of a family-based intervention for dietary intake and physical activity behaviour modification, it was found that energy intake was 41% lower than energy expenditure among obese children [42].

High intake of energy-dense food with high levels of energy derived from fat or sugar and fewer servings of fruit and vegetables are associated with overweight and obesity. Therefore, potentially effective ways to reduce obesity among children are to increase fruit and vegetable intake and to decrease fat consumption [43]. The results of the present study showed that the consumption of dietary fibre improved in both groups, but the quantity was less than the recommended intake, which is 20.0 to 30.0 g per day [44]. The mean difference in dietary fibre intake for both groups were almost the same as those reported by Zalilah [45] for schoolchildren. Participants in IG may be aware of the importance of consuming fruits and vegetables, but the availability of those foods may affect their intakes. Parents claimed that they could not afford to buy fruit and to make sure fruit was available at home most of the time. In addition, the types and quantities of fruits sold in schools are also limited.

Implementation of SOC in nutrition counselling among obese children was effective in modifying the behaviours of fruit, vegetable and fat consumption. After the intervention, it was found that children at higher SOC (maintenance or action) consumed less fat than those at lower SOC, specifically precontemplation. This difference showed that enrolment in nutrition counselling increased knowledge or raised consciousness about nutrition. However, improvement in knowledge alone is not enough to change the behaviour of children in the preparation, implementation and maintenance of new health habits. Instead of merely delivering nutrition knowledge based on SOC of participants, involving participants in a group activity may potentially affect their dietary choices. Furthermore, acknowledging children’s fruit and vegetable preferences can also helpful in encouraging children to increase their intake. A local study reported that children favour certain fruits (apples and mangos) and vegetables (water spinach, carrots, spinach, long beans, cucumbers and cabbage) and prefer an attractive presentation [46].

According to a systemic review of *dietary interventions effective at increasing fruit and vegetable consumption among overweight children, Bourke* [47] *found that the success of the intervention was determined by the study design. A good study design must provide a holistic approach* that not only promotes healthy diets, nutritional education (to increase fruit and vegetable intake) and physical activity but also incorporates community support, changes school policies to promote healthy nutrition and physical activity participation and provides parents with nutritional education and support. Moreover, it would provide the opportunities and settings to make these changes, such as increasing the availability of fruit within school cafeterias.

Several limitations of this study need to be highlighted. Body composition can be accurately measured using a dual-energy X-ray absorptiometry (DXA) scan and other laboratory-based techniques that would enhance the results from the present study [48], but this was not feasible given resource and funding constraints. Bioimpedance analysis (BIA) is also a good alternative method to DXA, but it cannot be used for children under 10 years old [49]. Owing to the limitation of the equipment, skinfold thickness at two sites (triceps and subscapular) was used to calculate the fat percentage, which may not give accurate results. To increase accuracy, we conducted the measurement twice, and the researcher performed the measurement to minimize inter-observer variation. Second, the use of a three-day food record in this study was subjected to under-reporting (~ 50%), despite the use of various measures to minimize its occurrence. Nevertheless, the reported intakes could also reflect the actual intakes of the obese children in attempts to reduce caloric intakes. Finally, subjective (e.g., self-report questionnaire) and objective (e.g., heart rate monitor, accelerometer and pedometer) measures of physical activity have been utilized in studies involving obese children. In the present study, a self-report questionnaire was used for ease of administration. However, as with dietary reporting, the use of self-reported physical activity may produce bias, particularly relating to social desirability. Unlike intention to treat analysis, the use of per protocol analysis in the present study could introduce bias to the study findings.

The present study showed the importance of delivering information or conducting programmes according to the SOC of the targets. The SOC approach can help policymakers make the best use of scarce resources by targeting nutrition programmes to match individuals’ readiness to change. One way to do this is to set eligibility requirements for more intensive nutrition programmes, which may be designed to help people set goals, solve problems, and implement new practices.

Furthermore, health care professionals in the primary care setting, especially family physicians, often have a long, trust-based relationship with patients. Therefore, they are also in an ideal position to help obese children and families through the slow, incremental process of achieving a healthy weight. Physicians can utilize the contents of the stage-based lifestyle modification, which guide them to provide basic advice on fat, fruit and vegetable intake, as well as physical activity, according to the patient’s stage of change.

## Conclusion

Childhood obesity is a recognized health related, and its prevalence is growing rapidly. The main treatment used to manage obesity in children is lifestyle modification encompassing changes in dietary intake and physical activity. The findings from the present study suggest that stage-based lifestyle modifications may offer an effective treatment to maintain weight and WC as well as increase physical activity for the management of childhood obesity.

## Abbreviations

BIA: Bioimpedance Analysis
BMI: Body mass index
CG: Control group
DXA: Dual-energy X-ray absorptiometry
IG: Intervention group
ITT: Intention-to-treat
NHMS: National Health and Morbidity Survey
NPG: Nutrition Practice Guideline
PAQ-C: Physical Activity Questionnaire for Older Children
SFA: Saturated Fatty Acids
SOC: Stages of Change
TTM: Transtheoretical Model
UPM: Universiti Putra Malaysia
WC: Waist circumference
WHO: World Health Organization
